# On-demand route discovery in a unicast manner

**DOI:** 10.1371/journal.pone.0204555

**Published:** 2018-10-01

**Authors:** Youngchol Choi, Hyun Jong Yang

**Affiliations:** 1 Marine ICT Research Division, Korea Research Institute of Ships and Ocean Engineering, Daejeon 34103, Republic of Korea; 2 School of Electrical and Computer Engineering, UNIST, Ulsan 44919, Republic of Korea; City University of Hong Kong, HONG KONG

## Abstract

While having high bandwidth-efficiency, the ad-hoc on-demand distance vector (AODV) routing protocol suffers from high signaling overhead due to route request (RREQ) messages flooding, especially when the node density and the number of connections are increased. In order to resolve this broadcast storm problem of the AODV in a high node density mobile ad-hoc network, we propose a geographical on-demand route discovery scheme. Assuming a known location of the destination, the RREQ of the proposed routing protocol is propagated in a *unicast* manner by employing a novel parsing mechanism for possible duplicate RREQs. The routing overhead of the proposed routing protocol is greatly robust to the node density change. We derive the node density required for the proposed routing protocol to keep the same connectivity as the AODV under the circumstance where the nodes are uniformly distributed. In addition, we present an imaginary destination consideration method to incorporate the uncertainty of the destination’s location due to mobility. Computer simulations show that the proposed scheme enables the RREQ propagation to cover 95% of the one-hop communication area centered at the originally known location of the destination without sacrificing the unicast feature.

## Introduction

The application of marine very high frequency (VHF) radio is now expanding from hand-held transceivers or safety purposes, such as automatic identification system (AIS), to high speed digital data communications [1–5]. Specifically, VHF data exchange (VDE) service, which is one of the key enablers for e-Navigation, is being actively discussed for standardization. The main traffic of maritime communication services is from many ships to a gateway located at the shore side. Therefore, the location of the destination is fixed and can be known to all the nodes. A ship can get its location using the global positioning system (GPS) which is one of the mandatory equipments of seagoing ships. Maritime mobile ad-hoc network (MANET) can provide cost-effective VDE services and may be an alternative for expensive satellite communication services. The most important features of the maritime MANET are high node density, many connections to a single destination, and limited bandwidth. In this network, the overall network performance is greatly influenced by routing overhead. If multi-hop communications are allowed in ultra dense networks (DenseNets) [6, 7], the DenseNets would be very similar to the maritime MANET. The network models of the vehicle-to-infrastructure communication of vehicular ad hoc networks (VANETs) [8–10] and underwater acoustic networks [11–14] are also analogous to the maritime MANET. Considering that the bandwidth is one of the most expensive resources in the MANET, it is important to minimize routing overhead to provide cost-effective high quality network services.

Reactive routing protocols are more attractive than table-driven approaches in the maritime MANET because of the frequent topology change and the limited bandwidth. Ad-hoc on-demand distance vector (AODV) [15, 16], the most popular one among reactive routing protocols, floods a route request (RREQ) message to find a valid path. This RREQ flooding causes unnecessary overhead that degrades the network performance such as packet delivery ratio and end-to-end latency. In addition, the success of RREQ broadcast suffers from the hidden node problem [17].

The broadcast storm problem of the AODV can be mitigated by employing an efficient multi-hop broadcast method, for example [17–27]. The aim of a multi-hop broadcast scheme is to make all the nodes hear the broadcast message with reduced rebroadcasts instead of flooding. Since a discovered route consists of a few nodes, the RREQ rebroadcasts by the nodes which are not selected as the route are necessary from a broadcast point of view but unnecessary overhead from a routing point of view. The number of these unnecessary RREQ rebroadcasts is increased as the network range increases. Therefore, although a near-optimal broadcast protocol is recently proposed [25], the routing overhead reduction resorting to a broadcast protocol is fundamentally limited by the network-wide dissemination of the RREQ. In [28–34], instead of network-wide search, the search zone is narrowed using some side information, and the principle of the broadcast protocol is applied to the narrowed zone for further reduction of routing overhead. However, the routing overhead reduction of [28–34] is also restricted by the fundamental limit of the broadcast protocols.

In order to remove redundant RREQs completely in a dense network, we propose a geographical AODV (GAODV) whose RREQ is propagated in a unicast manner. Our contributions in this paper are three-fold as follows:

- *Duplication control and passive acknowledgement*: Instead of the RREQ dissemination to all the nodes of the network or of a specific region, only one node among the one-hop neighbors of the RREQ sender is involved in the route discovery procedure of the GAODV. The number of the RREQ rebroadcasts of the GAODV is equal to the hop count from the source to the destination, and thus, the GAODV minimizes the RREQ rebroadcast. Especially in a dense network with heavy traffic, this minimized RREQ rebroadcast improves the route acquisition probability and reduces the route acquisition time, which leads to significant improvement of the packet delivery ratio and end-to-end latency performance. The key step of the GAODV is the parsing procedure of duplicate RREQs, which enables both the duplication control and the passive acknowledgement with respect to the RREQ rebroadcast. The passive acknowledgement relieves the hidden node problem induced by the RREQ broadcast.
- *Required node density*: The RREQ rebroadcast of the GAODV reduces the zigzag phenomenon of the discovered path, but this reduced zigzag and the elimination of redundant RREQs degrade the connectivity at the low node density because the amount of zigzag of a connectable path increases as the node density decreases. We draw the node density required for the GAODV to keep the same connectivity as the AODV.
- *Uncertainty of the destination’s location*: In order to alleviate the assumption of the fixed destination’s location, we present an imaginary destination method that initiates a route discovery to a virtual location on the line connecting the source and the destination. The optimum imaginary destination is derived in terms of the maximum speed of a node and the elapsed time since the last update of the destination’s location. The proposed imaginary destination method does not sacrifice the unicast feature of the GAODV and can cover 95% of one-hop communication area centered at the known location of the destination.

## 1 Related work

We restrict ourselves to the methods for the overhead improvement of the AODV. Interested readers are referred to [20, 35, 36] for the extensive survey on MANET routing protocols. Dynamic source routing (DSR) [37], a pioneering work of on-demand routing protocol for MANET, introduces for the first time the concept of the RREQ and the route-reply (RREP). In the DSR, each RREQ rebroadcast appends the address of the rebroadcast node to the RREQ packet. In other words, the RREQ includes overall path information before this RREQ packet reaches the current node. Therefore, the RREQ packet length is proportional to the hop count. Also, data packets include the information on the whole route to be traversed. The AODV follows the same principle of the DSR, but the next-hop to reach the destination, instead of the overall path information of the DSR, is provided by the local routing table. In the AODV, the routing loop is avoided by employing the sequence number technique of the Destination-Sequenced Distance-Vector (DSDV) [38].

Although the routing overhead of the AODV is significantly smaller than that of the table driven routing protocols, the broadcast storm problem due to the network-wide flooding of the RREQ may cause severe performance degradation in band-width limited applications as the number of concurrent route requests or topology change rate increases in a high node density scenario.

Extensive effort has been put to improve the overhead of the AODV. The RREQ flooding efficiency can be improved by clustering [18], probabilistic rebroadcast [17, 19, 20], selective rebroadcast based on the coverage area estimation [17, 21, 22] or the neighbor knowledge [23–25], and probabilistic rebroadcast based on the neighbor knowledge [26, 27]. Numerical performance comparisons of broadcast protocols can be found in [39]. The approaches based on neighbor knowledge show the best performance in terms of the number of retransmitting nodes. However, periodic “HELLO” packets are required to learn and keep the information of neighbors, which cannot be affordable in bandwidth-limited applications with high node density. Recently, application-specific methods are proposed to decrease redundancy in delay tolerant networks [40], pedestrian ad-hoc networks [41], and VANET [42]. In general, these efficient broadcast methods make an attempt for the RREQ to be disseminated to the whole network with only partial flooding instead of full flooding. Redundant RREQ rebroadcasts, which are not served as the route, are inevitable for the network-wide coverage of the RREQ.

The RREQ propagation is guided to the general direction of the destination with the aid of some side information in [28–34]. The flooding of the location-aided routing (LAR) protocol [28] is restricted to a specific region determined by the speed and physical locations of the source and the destination. The redundancy of the LAR can be more reduced by counting duplicate RREQs [29]. The regional gossip routing [30] takes the benefit of the probabilistic and the geographic methods simultaneously, in which only the nodes inside an ellipse determined by assuming the locations of the source and the destination as foci are involved in a gossip-based route recovery procedure. The mobility can be positively exploited to narrow the search zone without physical location information as shown in [31–34]. The FRESH [31] uses the following “time-distance correlation” principle induced by the mobility: a node that encounters the destination more recently is nearer to that destination, that is, “encounter age” is lower. The routing overhead of the FRESH is reduced by a successive smaller search scheme that finds the nearest anchor node having smaller encounter age. This principle of [31] is also employed in [32, 33]. Unlike the constant gossiping probability of [19, 30], the gossiping probability of [33] is modified such that the search zone is steered towards the general direction of the destination without an external positioning service. In [34], the distance between a pair of nodes is estimated explicitly and the gossiping probability is adaptively determined based on the estimated distance. However, sufficient warm-up period is essential to reach the steady state in the protocols of [31–34] because the required information is extracted from the past contact history between nodes.

Seemingly, the operation of the proposed GAODV is similar to the relaying node selection procedure of the GeRaF [43, 44] because a data packet is delivered to the destination without broadcast redundancy in the GeRaF using the location information of the sender and the destination. But the relaying node selection of the GeRaF is done in a cross-layer framework, and the details of the GeRaF should be modified according to the change of medium access control (MAC) protocol. Furthermore, the iterative nature of the collision resolution step requires more additional message changes as the increase of the node density, which also increases the required time for the relaying node selection that corresponds to the route acquisition time. Therefore, the overhead for the relaying node selection can be significantly increased in a high density scenario. On the other hand, the proposed GAODV inherits MAC-independence from the AODV and does not require any additional collision resolution mechanism.

## 2 Geographical AODV

We call the RREQ of the GAODV geographical RREQ (GRREQ) to distinguish it from the RREQ of the AODV. The GRREQ, initiated by the source *S* to the destination *D*, and transmitted by the sender *T*, is denoted by GRREQ(*T, S, D*). We assume that all the nodes know their own locations, and that the location of the destination is known by all the nodes in the network. Generally, these two assumptions are common in routing protocols that use location information. In addition, we assume that the location of the sender is embedded in the GRREQ packet. The distance between the two nodes *N_1_* and *N*_2_ is denoted by d(*N*_1_, *N*_2_). The radius of one-hop wireless communication range is denoted by *R*.

### 2.1 Selective rebroadcast of route request

**Fig 1:**
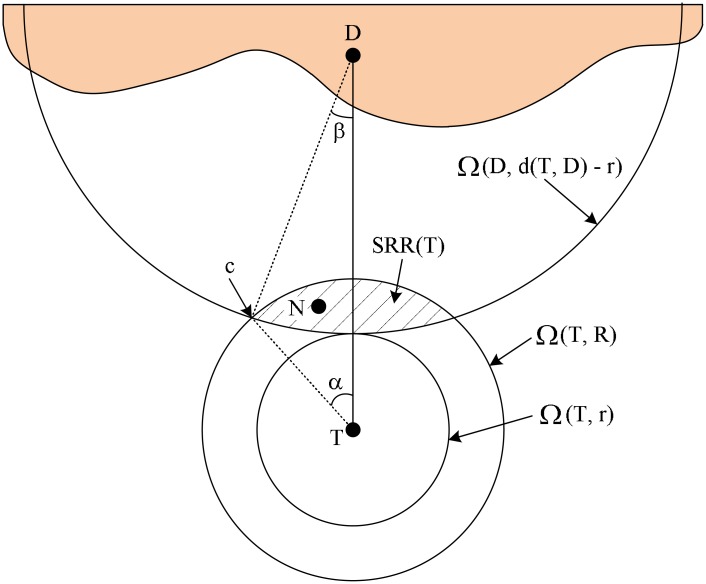
Graphical illustration of the SRC and the SRR of the GRREQ.

First of all, the candidates to be involved in the route discovery procedure are limited by a small portion of the one-hop communication area of the sender, which is determined by the locations of the sender and the destination. If a node *N* receives the GRREQ(*T, S, D*), *N* is involved in the route recovery procedure by relaying GRREQ(*N, S, D*) only if *N* satisfies following condition,

$$\begin{array}{c} \text{d}(N,D)<\text{d}(T,D)-r, \end{array}$$ (1)

where *r* is a non-negative constant smaller than *R*. Otherwise, *N* discards the received GRREQ. We call (1) selective relaying condition (SRC), and refer the region satisfying SRC to as selective relaying region (SRR). The SRR created by *T* is denoted by SRR(*T*). The concept of the SRC and the SRR is illustrated in Fig 1. SRR(*T*) is the intersection of Ω(*D*, d(*T*, *D*) − *r*) and Ω(*T*, *R*), where Ω(*X*, *x*) is an open ball of radius *x* centered at the location of *X*. In Fig 1, SRR(*T*) is the region filled with deviant crease lines. We denote the area of SRR(*T*) as A(SRR(T)). Intuitively, as shown in Fig 1, SRR is chosen such that the route is geometrically close to the line that connects the sender and the destination. In AODV, all nodes within Ω(*T*, *R*) relay RREQ only once. On the other hand, the rebroadcast of GRREQ is geographically restricted to SRR(*T*). This geographical restriction of the GRREQ rebroadcast can reduce the routing overhead of the AODV because A(SRR(T)) is smaller than the area of Ω(*T*, *R*).

The value of *r* determines A(SRR(T)). We can get A(SRR(T)) using elementary Euclidean geometry as follows,

$$\begin{array}{cc} A(\text{SRR}(T)) & =R^{2}\left(\alpha -\sin\alpha \cos\alpha \right)+{\left(d\left(T,D\right)-r\right)}^{2}\\ & \;\times (\beta -\sin\beta \cos\beta ). \end{array}$$ (2)

If an intersection point of ∂Ω(*D*, d(*T*, *D*) − *r*) and ∂Ω(*T*, *R*) is denoted by *c*, where ∂Ω(*X*, *x*) is the boundary of Ω(*X*, *x*), *α* and *β* are ∠*DTc* and ∠*TDc*, respectively. If *r* is given, A(SRR(T)) can be obtained by computing trigonometric functions of *α* and *β* from the law of cosines. It is clear that A(SRR(T)) is a monotonically decreasing function of *r*. Therefore, *r* should be sufficiently small to guarantee the connectivity of the GAODV for a given node density. This connectivity analysis will be detailed in Section 2.4.

### 2.2 Redundancy control

Routing overhead can be reduced to a certain degree by the selective rebroadcast discussed in Section 2.1 because the area of the SRR is smaller than the entire one-hop communication area. However, this regional partial flooding still incurs many unnecessary redundant GRREQs which serve as overhead. Furthermore, it is not easy to control adaptively A(SRR(T)) for the minimization of routing overhead, while the connectivity is always guaranteed. The duplicate GRREQ, like the duplicate RREQ in the AODV, has the same GRREQ ID and the source address as the firstly received GRREQ. In the GAODV, duplicate GRREQs are rather positively utilized to control the redundancy of the GRREQ by parsing those duplicate GRREQs, which are generally discarded in the protocols that follow the principle of the AODV.

Let us consider the rebroadcast of the GRREQ on the reception of GRREQ(*T, S, D*). Note that in the AODV, the RREQ ID and the source address of the RREQ received for the first time are maintained to check duplicate RREQs. In order to identify a duplicate GRREQ, we use the hop count field of the GRREQ as well as the GRREQ ID and the source address, referred to as information to parse the duplicate GRREQ (abbreviated as IPD, which is a tuple composed of the GRREQ ID, the source address, and the hop count). We assume that GRREQ(*T, S, D*) is the firstly received GRREQ packet to all the nodes within SRR(*T*). If a node within SRR(*T*) receives GRREQ(*T, S, D*), then the IPD with respect to GRREQ(*T, S, D*) is recorded. Assume that *F* located within SRR(*T*) is the first node that rebroadcasts GRREQ(*T, S, D*). If another node within SRR(*T*) receives GRREQ(*F, S, D*) (which is the rebroadcast of GRREQ(*T, S, D*)) after the reception of GRREQ(*T, S, D*), it can be decided whether the received duplicate GRREQ(*F, S, D*) is relayed by a node within SRR(*T*) or not. The key observation is that the hop count of GRREQ(*F, S, D*) is larger than the hop count of GRREQ(*T, S, D*) by one. In short, if any node *N* within SRR(*T*) receives a duplicate GRREQ whose hop count is larger than that of GRREQ(*T, S, D*) by one, then *N* decides that some node within SRR(*T*) is already relayed GRREQ(*T, S, D*) and does not broadcast GRREQ(*N, S, D*) to avoid unnecessary redundant GRREQ although *N* is a candidate for the rebroadcast of GRREQ(*T, S, D*). In this way, the GRREQ can be propagated to the destination in a unicast manner without redundant GRREQs. We call this hop count increase by one redundancy control condition (RCC).

The value of *r* in the SRC should be determined by taking into account of the node density. However, it is not necessary to optimize *r* for the minimization of routing overhead because redundant GRREQs can be effectively controlled by the RCC. Therefore, the routing overhead of the proposed GAODV is greatly robust to the node density change.

### 2.3 Passive acknowledgement

Parsing duplicate GRREQs facilitates passive acknowledgment as well as redundancy control. Let us consider a GRREQ propagation chain: GRREQ(*T*_0_, *S, D*), GRREQ(*T*_1_, *S, D*), ⋯, GRREQ(*T_i_, S, D*), ⋯. The GRREQ(*T_i_, S, D*) is the rebroadcast of the GRREQ(*T*_*i*−1_, *S, D*). *T_i_* is located within SRR(*T*_*i*−1_) for all *i*, and *T*_0_ is the source S. *T_i_* records the IPD with respect to the received the GRREQ(*T*_*i*−1_, *S, D*). Therefore, the hop count of the IPD at *T_i_* is *i* − 1. If *T*_*i*+1_ broadcasts the GRREQ(*T*_*i*+1_, *S, D*), then *T_i_* also receives the GRREQ(*T*_*i*+1_, *S, D*) which is a duplicate of the GRREQ(*T*_*i*−1_, *S, D*). In *T_*i*_*, this event can be regarded as passive acknowledgement of the GRREQ(*T_i_, S, D*) because the GRREQ(*T*_*i*+1_, *S, D*) is the rebroadcast of the GRREQ(*T_i_, S, D*) induced by the GRREQ(*T*_*i*−1_, *S, D*). Considering that the hop count of the GRREQ(*T*_*i*+1_, *S, D*) is *i* + 1, the essential constraint for *T_i_* to recognize a duplicate GRREQ as the passive acknowledgement of the GRREQ(*T_i_, S, D*) is that the hop count of the IPD at *T_i_* is larger than that of the duplicate GRREQ by two. We call this hop count increase by two passive acknowledgement condition (PAC).

The condition for the passive acknowledgement at the source is different with the PAC at an intermediate node except for the source. If *S* receives the GRREQ(*T*_1_, *S, D*), the hop count of which is “1”, *S* decides that the GRREQ initiated by *S* is successfully rebroadcasted by a node within SRR(*S*). Considering that the hop count of the GRREQ(*T*_0_, *S, D*) is “0”, this passive acknowledgement scenario of the source may appear to be similar with RCC. But the reason for this different passive acknowledgement condition at the source is that the information for the firstly received GRREQ is not available at the source. It will be helpful to recall that the subjects of the duplication check at the source and an intermediate node are the GRREQ transmitted by itself and the firstly received GRREQ, respectively. Although the hop count condition of PAC is dependent on whether the node is the source or not, we use the same terminology PAC at both intermediate node and the source because the use of PAC can be clarified contextually.

### 2.4 Connectivity

The connectivity is defined as the reachability under ideal MAC and physical layer. Any method to improve the routing overhead of the AODV always suffers from the connectivity degradation. The only disadvantage of the GAODV is the connectivity degradation. For the connectivity analysis of the GAODV, it is assumed that nodes are deployed with uniform distribution in a stationary scenario. Let us revisit Fig 1 to compute the probability for the connectivity to be broken at node *T*. We define *ϵ* as $\frac{A(\text{SRR}(T))}{\pi R^{2}}$. The parameter *ϵ* indicates how much proportion of the one-hop wireless communication area *πR*^2^ is involved in the route discovery procedure. The node density *ρ* is defined as the average number of nodes in one-hop wireless communication area *πR*^2^. We only consider *ρ* of integer value for convenience, but the extension to the case with real *ρ* is straightforward. On average there are *ρ* nodes in the one-hop communication area of node *T*, and the probability for all *ρ* nodes to be located outside SRR(*T*) is (1 − *ϵ*)^*ρ*^. There is at least one node in SRR(*T*) with a probability of

$$\begin{array}{c} p(\epsilon ,\rho )=1-{\left(1-\epsilon \right)}^{\rho }=-\sum_{k=1}^{\rho }(\begin{array}{c} \rho \\ k \end{array}){\left(-\epsilon \right)}^{k}. \end{array}$$ (3)

Therefore, the connectivity is not broken at an intermediate rebroadcast node *T* with a probability of *p*(*ϵ*, *ρ*). In Eq (3), *p*(*ϵ*, *ρ*) is a function of two variables *ϵ* and *ρ*, but we claim that *p*(*ϵ*, *ρ*) is approximately dependent on the product of *ϵ* and *ρ* as described in the following proposition.

**Proposition 1**. *Assume that ϵ*_1_*ρ*_1_ = *ϵ*_2_*ρ*_2_ = *C for a constant C, then*

$$\begin{array}{c} |p\left(\epsilon_{1},\rho_{1}\right)-p\left(\epsilon_{2},\rho_{2}\right)|\propto \frac{1}{\rho_{1}}. \end{array}$$ (4)

*Proof*: From Eq (3), we can get

$$\begin{array}{l} p\left(\epsilon_{1},\rho_{1}\right)-p\left(\epsilon_{2},\rho_{2}\right)=\left\{\epsilon_{1}\rho_{1}-\epsilon_{2}\rho_{2}\right\}\\ -\left\{\frac{\rho_{1}\left(\rho_{1}-1\right)}{2}\epsilon_{1}^{2}-\frac{\rho_{2}\left(\rho_{2}-1\right)}{2}\epsilon_{2}^{2}\right\}\\ +\left\{\frac{\rho_{1}\left(\rho_{1}-1\right)\left(\rho_{1}-2\right)}{6}\epsilon_{1}^{3}-\frac{\rho_{2}\left(\rho_{2}-1\right)\left(\rho_{2}-2\right)}{6}\epsilon_{2}^{3}\right\}+\cdots . \end{array}$$ (5)

The first term of the right hand side (RHS) of Eq (5) is eliminated by assumption. The second term of the RHS of Eq (5) can be rewritten by

$$\begin{array}{c} |-\frac{\left(\rho_{1}^{2}\epsilon_{1}^{2}-\rho_{2}^{2}\epsilon_{2}^{2}\right)-\left(\rho_{1}\epsilon_{1}^{2}-\rho_{2}\epsilon_{2}^{2}\right)}{2}|=|\frac{\rho_{1}\epsilon_{1}\left(\epsilon_{1}-\epsilon_{2}\right)}{2}|\propto \frac{C^{2}}{2\rho_{1}}. \end{array}$$ (6)

In Eq (6), the highest degree component $\left(\rho_{1}^{2}\epsilon_{1}^{2}-\rho_{2}^{2}\epsilon_{2}^{2}\right)$ is cancelled out by assumption, and the second term of the RHS of Eq (5) becomes inversely proportional to the node density. Similarly, the remained terms of the RHS of Eq (5) are also inversely proportional to the node density. Therefore, we can conclude that the magnitude of *p*(*ϵ*_1_, *ρ*_1_) − *p*(*ϵ*_2_, *ρ*_2_) is inversely proportional to the node density.

The product of *ρ* and *ϵ* is the average number of nodes in SRR(*T*). Therefore, Proposition 1 tells us that the probability for the GRREQ propagation to be broken at an intermediate node *T* is dependent on the average number of nodes in SRR(*T*).

**Fig 2:**
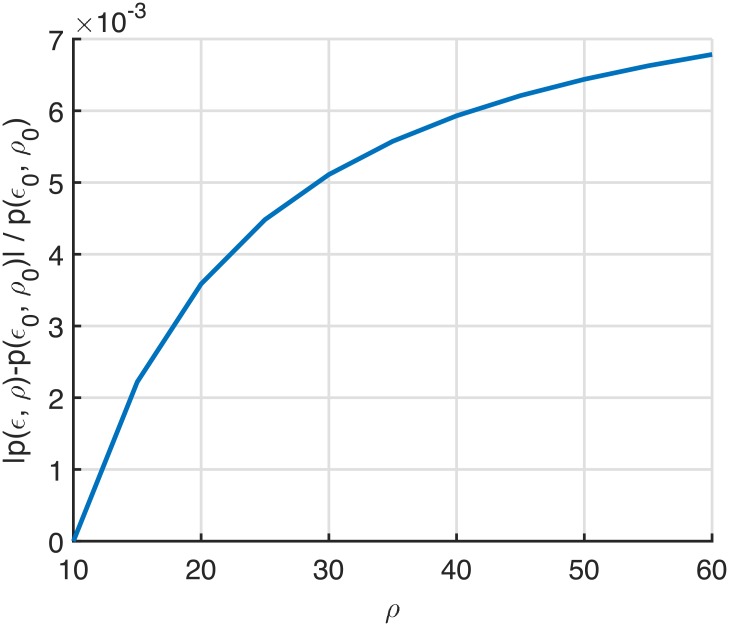
Numerical example for the verification of *ϵρ* dependency of *p*(*ϵ*, *ρ*).

Fig 2 validates Eq (4) as a numerical example, showing $\frac{\left|p\left(\epsilon ,\rho \right)-p\left(\epsilon_{0},\rho_{0}\right)\right|}{p\left(\epsilon_{0},\rho_{0}\right)}$ for increasing *ρ*, where (*ρ*_0_, *ϵ*_0_) = (10, 0.45). The value of *ϵ* is determined such that *ϵρ* = *ϵ*_0_ *ρ*_0_ is satisfied. It can be seen from Fig 2 that the value of $\frac{\left|p\left(\epsilon ,\rho \right)-p\left(\epsilon_{0},\rho_{0}\right)\right|}{p\left(\epsilon_{0},\rho_{0}\right)}$ is less than 0.7% in spite of six-times increased *ρ*, which clearly shows *ϵρ* dependency of *p*(*ϵ*, *ρ*). In addition, the monotonically decreasing slope of Fig 2 shows the validity of Eq (4).

The instantaneous property for the connectivity can be explained by Eq (3), but it is not straightforward to analyze the end-to-end connectivity using Eq (3) due to the randomness of the GRREQ travel path. We derive an analytic formula for the end-to-end connectivity of the GAODV in the following proposition.

**Proposition 2**. *Consider a* (*K* + 1)-*hop GRREQ propagation chain: GRREQ(T*_0_, *S, D), GRREQ(T*_1_, *S, D*), ⋯, *GRREQ(T_k_, S, D*), ⋯, *GRREQ(T_K_, S, D) where T_k_ is located within SRR(T*_*k*−1_) *for all k and T*_0_ *is the source S*. *The connectivity of the* (*K* + 1)-*hop GRREQ propagation chain is given by*

$$\begin{array}{cc} & \int_{x_{K-2}-R}^{x_{K-2}-r}\cdots \int_{x_{k-1}-R}^{x_{k-1}-r}\cdots \int_{x_{0}-R}^{x_{0}-r}\prod_{k=1}^{K}p\left(\epsilon \left(x_{k-1}\right),\rho \right)\\ & \;\times f_{k}\left(x_{k}\right)dx_{1}\cdots dx_{k}\cdots dx_{K-1}, \end{array}$$ (7)

*where*

$$\begin{array}{c} f_{K}\left(x_{K}\right)=1, \end{array}$$ (8)

$$\begin{array}{c} f_{k}\left(x_{k}\right)=\frac{2\arccos(\frac{x_{k}^{2}+x_{k-1}^{2}-R^{2}}{2x_{k}x_{k-1}})x_{k}}{\pi R^{2}\epsilon \left(x_{k-1}\right)},k\leq K-1, \end{array}$$ (9)

*and ϵ*(*x_k_*) *is the value of ϵ at the distance of x_k_*.

**Fig 3:**
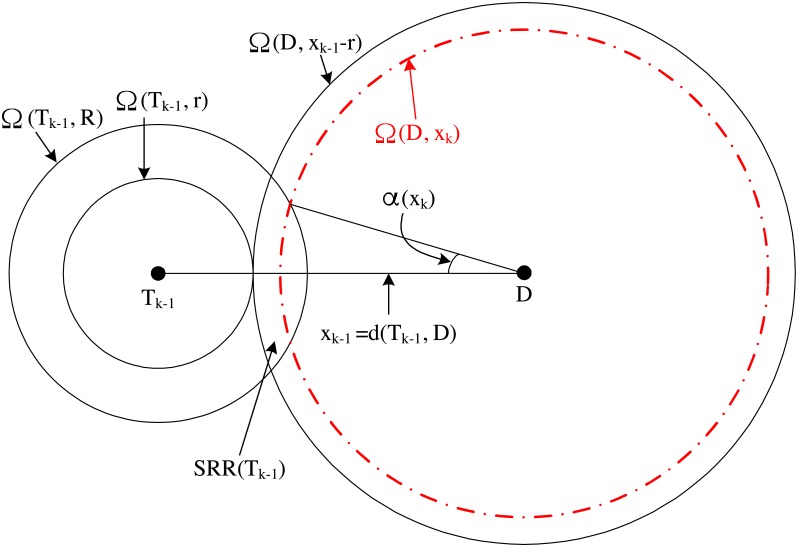
Computation of *f*_*k*_(*x*_*k*_) from the broadcast of GRREQ(T_*k*−1_, S, D) in a (*K* + 1)-hop GRREQ propagation chain.

*Proof*. Fig 3 shows the broadcast of GRREQ(*T*_*k*−1_, *S, D*). *T_*k*_* is chosen randomly among the nodes within SRR(*T*_*k*−1_). Therefore, d(*T_k_, D*) is a random variable. The probability density function (PDF) of d(*T_k_, D*), denoted by *f*_*k*_(*x*_*k*_), is dependent on the arc length determined by the intersection of ∂Ω(D, *x*_*k*_) and SRR(*T*_*k*−1_). This arc length is 2*α*(*x*_*k*_)*x*_*k*_, and *α*(*x*_*k*_) can be obtained from the law of cosines as follows

$$\begin{array}{c} \cos\alpha \left(x_{k}\right)=\frac{x_{k}^{2}+x_{k-1}^{2}-R^{2}}{2x_{k}x_{k-1}}. \end{array}$$ (10)

Now, we get

$$\begin{array}{c} f_{k}\left(x_{k}\right)=\frac{2\alpha \left(x_{k}\right)x_{k}}{\int_{x_{k-1}-R}^{x_{k-1}-r}2\alpha \left(x_{k}\right)x_{k}dx_{k}}. \end{array}$$ (11)

The denominator of Eq (11) is the area of SRR(*T*_*k*−1_), and hence Eq (11) can be rewritten as follows

$$\begin{array}{c} f_{k}\left(x_{k}\right)=\frac{2\alpha \left(x_{k}\right)x_{k}}{\pi R^{2}\epsilon \left(x_{k-1}\right)}. \end{array}$$ (12)

*T_K_* is a one-hop neighbor of D because GRREQ(*T_K_, S, D*) is the last rebroadcast. Therefore, if we define *f*_*K*_(*x*_*K*_) as shown in Eq (8), we get Eq (7) by computing (*K* − 1)-dimensional integration.

**Fig 4:**
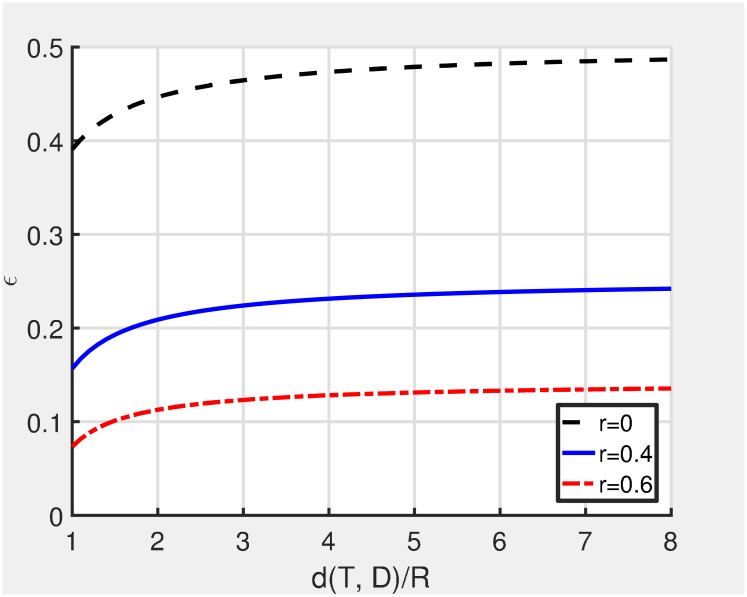
*ϵ* is a function of *r* and the distance from the sender to the destination.

**Fig 5:**
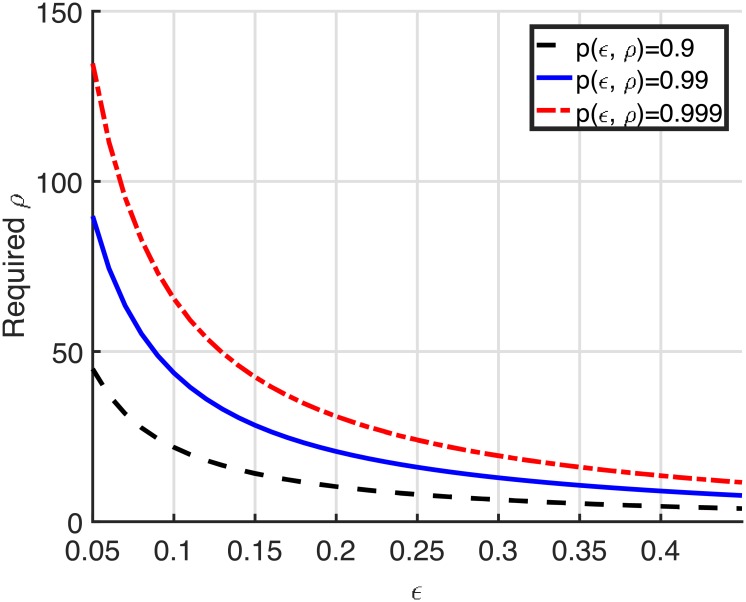
Required node density to achieve *p*(*ϵ*, *ρ*) of 0.9, 0.99, and 0.999.

It is difficult to compute Eq (7) because the random variable *x*_*k*_ is a non-linear function of random variables *x*_*k*−1_, *x*_*k*−2_, ⋯, *x*_1_. Instead of direct computation of Eq (7), we give insights for the connectivity by observing the trend of *ϵ*. The value of *ϵ* is dependent on *r* and the distance from the sender to the destination (d(*T, D*)) as shown in Fig 4. The horizontal axis of Fig 4 is d(*T, D*)/*R*. The value of *ϵ* is a monotonically increasing function of the distance and converges to an asymptotic limit rapidly as the distance is increased by more than 2. The connectivity is the product of *p*(*ϵ*, *ρ*) of all the GRREQ rebroadcasts, and *p*(*ϵ*, *ρ*) decreases as the GRREQ rebroadcast node becomes closer to the destination. Therefore, *p*(*ϵ*, *ρ*) at the distance between 1 and 2 is dominant in the connectivity, which may provide a certain predictability for the required node density to achieve a given connectivity. Fig 5 depicts the required node density to achieve *p*(*ϵ*, *ρ*) of 0.9, 0.99, and 0.999. For example, the GAODV with *r* = 0.6 has *ϵ* of 0.0732 at the distance of *R*. In this case, the required node density values to achieve *p*(*ϵ*, *ρ*) of 0.9 and 0.99 are 30.2902 and 60.5804, respectively, and thus we expect that the required node density of the GAODV with *r* = 0.6 for the connectivity of 0.9 and 0.99 may be around 30 and 60, respectively. Similarly, the required node density of the GAODV with *r* = 0.4 for the connectivity of 0.9 and 0.99 may be around 14 and 27, respectively. If we approximate the AODV as the GAODV with *r* = 0 which has *ϵ* of 0.3910 at the distance of *R*, the required node density of the AODV for the connectivity of 0.9 and 0.99 may be around 5 and 9, respectively. Note that Fig 5 becomes the exact connectivity if the hop count of a discovered route is two.

### 2.5 Uncertainty of the destination’s location

**Fig 6:**
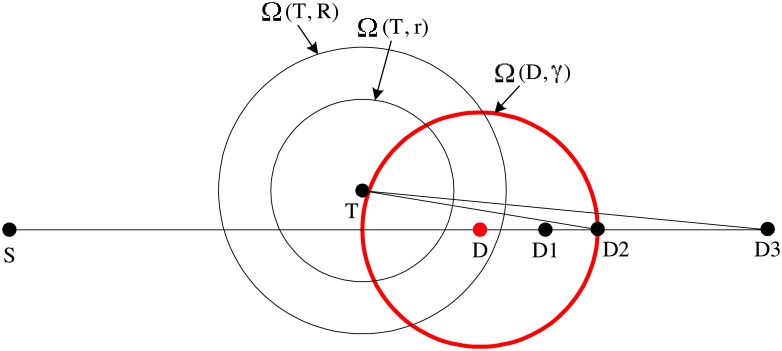
Imaginary destination method.

It is assumed in the GAODV that the destination is fixed at the location known to all the nodes. We alleviate this assumption for broader applicability of the GAODV. We assume that the speed of a node does not exceed *v*_*max*_. The mobility radius of the destination, denoted by *γ*, is defined by

$$\begin{array}{c} \gamma =v_{max}\Delta t, \end{array}$$ (13)

where Δ*t* is the elapsed time since the last update of the destination’s location. Then, the destination must be contained in Ω(*D*, *γ*). Note that Ω(*D*, *γ*) is a possible location of *D* derived on the assumption that *D* will travel at maximum speed. Our goal is to make GRREQ rebroadcasts cover Ω(*D*, *γ*) as much as possible without sacrificing the unicast feature of the GRREQ propagation. To this end, instead of the known location of the destination, we use an imaginary location on the line connecting the source *S* and the destination *D*, as shown in Fig 6. For convenience, we denote a straight line which passes through *X* and *Y* as Ψ(*X, Y*). Let us consider the route discovery to the known location of *D*. As shown in Fig 6, if the known location of *D* is in SRR(*T*), the GRREQ propagation stops at *T*. In this case, if *D* moves to a point within Ω(*D*, *γ*)-Ω(*T*, *R*), the GAODV may fail to find the route from *S* to *D*. However, for example, if we use *D1* instead of *D*, a node of SRR(*T*) rebroadcasts GRREQ(*T, S, D1*) because *D1* is out of Ω(*T*, *R*), and this additional rebroadcast can provide an extra coverage.

Since the the real destination after Δ*t* can be located at any point within Ω(*D*, *γ*), we propose to artificially set another destination, referred to as the imaginary destination, and make the GAODV routing pursue the imaginary destiation, not *D*. The aim is two-fold: 1) to make the real destination within Ω(*D*, *γ*) listen to the GRREQ relayed while it is routed to the imaginary destination, and 2) to shorten the routing path towards the imaginary destination. It is immediate from Fig 6 that a large portion of the area within Ω(*D*, *γ*) can be covered if the GRREQ routing is propagated along the diameter line of Ω(*D*, *γ*). In addition, to shorten the routing path, we propose to choose the imaginary destination to be the far side intersection point of Ω(*D*) and Ψ(*S, D*), e.g, *D*2 in Fig 6. Note that *D*1 and *D*3 in Fig 6 are not good options for the imaginary destination, because a large portion of the area within Ω(*D*, *γ*) cannot be covered for both cases.

## 3 Implementation of the GAODV

The GAODV is implemented by the modification of RREQ-related functions of the AODV in QualNet ver. 5.1. A detailed procedure of the GAODV implementation is given in what follows.

### 3.1 Message format of GRREQ

The GRREQ message format follows the RREQ message format of the AODV except for the following added fields,

- Selective flood (SF): This one-bit flag determines whether the GRREQ is selectively flooded (‘TRUE’) or not (‘FALSE’).
- Selective rebroadcast region depth (SRRD): SRRD is to control the area of the SRR when route discovery fails.
- Location of the sender (LoS) and location of the destination (LoD)

Although we choose sufficiently small *r* for the high connectivity, route discovery may still fail due to the imperfect connectivity. In this case, the GRREQ can be re-initiated with the decrease of *r* as follows

$$\begin{array}{c} r=r_{0}-\text{SRRD}\times r_{\Delta }, \end{array}$$ (14)

where *r*_Δ_ is the step size. Our suggestion is the SRRD of 3-bit. The SRRD field size should be sufficiently large for the fine adjustment of *r*, which means the increases of the GRREQ packet size. The SRRD field size should be determined by considering the requirements of target application. The reserved field of the RREQ of the AODV is large enough to put the SRRD and the SF. The size of the LoS and the LoD is dependent on the required precision of location. Most of the time 32-bits quantization is sufficient because the GAODV does not require accurate location. For example, if we assign 6/6/4-bit integer to the last one digit of degrees, minutes, and seconds, respectively, this quantization of the GPS coordinates can cover approximately 1,000 *km* × 1,000 *km*, in which the quantization error between two points is less than 100 m. Maritime VHF communication range is typically several tens of kilometers, and the quantization error normalized by the communication range is less than 1%. Therefore, 32-bits quantization for the LoS and the LoD is sufficient for maritime VHF communication networks. The RREQ packet size of the AODV is 192-bits in RFC 3561, and thus, the additional overhead of the GRREQ due to newly added fields is about 1/3 of that of the AODV.

### 3.2 Origination of the GRREQ at the source

**Fig 7:**
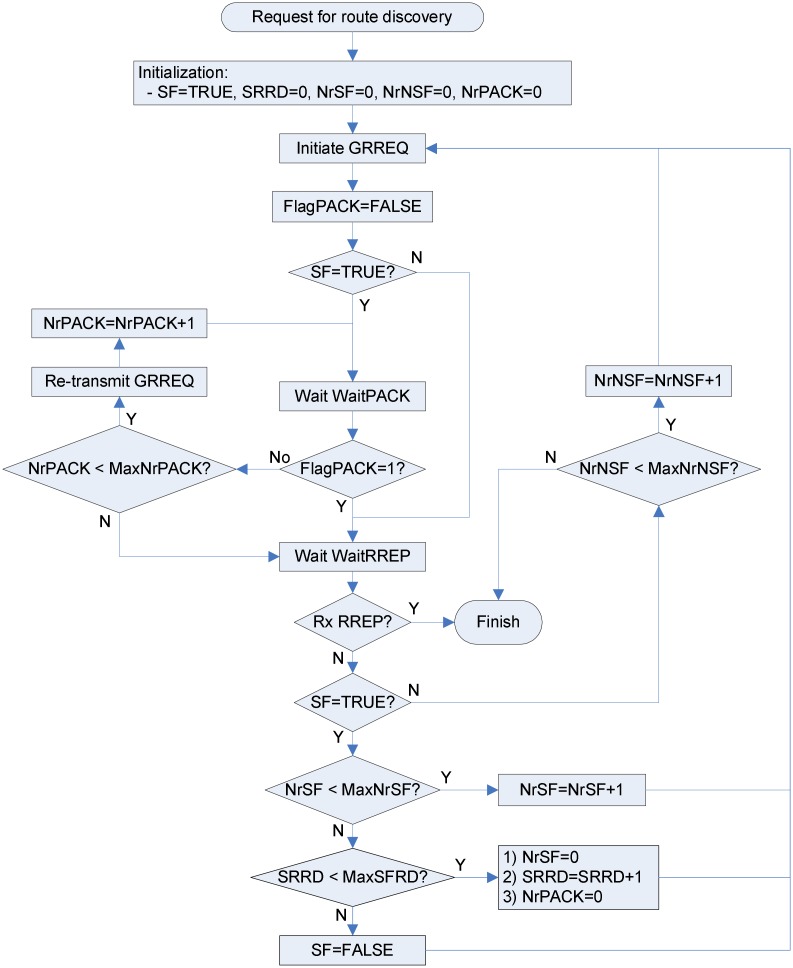
Flowchart for the RREQ initiation.

The flowchart for the GRREQ initiation at the source is shown in Fig 7. Terms used in Fig 7 are given below:

- FlagPACK: This one-bit flag is set to TRUE if the source receives a duplicate GRREQ with the hop count of ‘1’.
- NrSF: Number of retries with SF = TRUE.
- NrNSF: Number of retries with SF = FALSE.
- NrPACK: Number of the GRREQ re-transmissions.
- MaxNrSF: Maximum NrSF.
- MaxNrNSF: Maximum NrNSF.
- MaxNrPACK: Maximum NrPACK.
- MaxSRRD: Maximum SRRD.
- WaitPACK: Waiting time for the PAC to be fulfilled. WaitPACK corresponds to twice the node traversal time.
- WaitRREP: Waiting time for the RREP to arrive.

NrSF, NrNSF, NrPACK, and FlagPACK are variables associated with a specific GRREQ identified uniquely by the source address and the GRREQ ID. MaxNrSF, MaxNrNSF, MaxNrPACK, MaxSRRD, WaitPACK, and WaitRREP are global constants irrelevant to a specific GRREQ. If a request for route discovery is occurred, SF is set to TRUE and SFRD, NrSF, NrNSF, and NrPACK are initialized to zero. Then the source generates and disseminates the corresponding GRREQ. After waiting for WaitPACK, if the SF is TRUE and FlagPACK is FALSE, the source re-transmits the GRREQ up to MaxNrPACK-times. It is worthy to note that the re-transmission (ReTx) due to unsatisfied passive acknowledgement is not the retry that causes additional network-wide propagation of the GRREQ. The GRREQ ID is not increased by this ReTx. If the GRREQ is re-transmitted in spite of the successful rebroadcast by a node in SRR(S), e.g., if the passive acknowledgement is not recognized because of the temporarily bad wireless channel condition, this ReTx is discarded by the RCC at all one-hop neighbors of the source. Therefore, the influence of the ReTx is limited to one-hop neighbors of the source. The ReTx reduces route discovery failure (RDF) due to the temporary degradation of the physical communication channel such as fading.

If the FlagPACK is set to TRUE, then checks the RREP. After waiting for WaitRREP, if the RREP is not received and the SF is TRUE, the GRREQ is re-initiated up to MaxNrSF-times for each SRRD. If route discovery fails until SRRD is greater than MaxSRRD, the GAODV gives up the selective rebroadcast mechanism by setting the SF to FALSE and returns to the conventional AODV. This fall-back mechanism is necessary to deal with the failure of the unicast-like operation. For example, the destination may be out of the coverage of the imaginary destination method.

### 3.3 Parsing the received GRREQ

**Fig 8:**
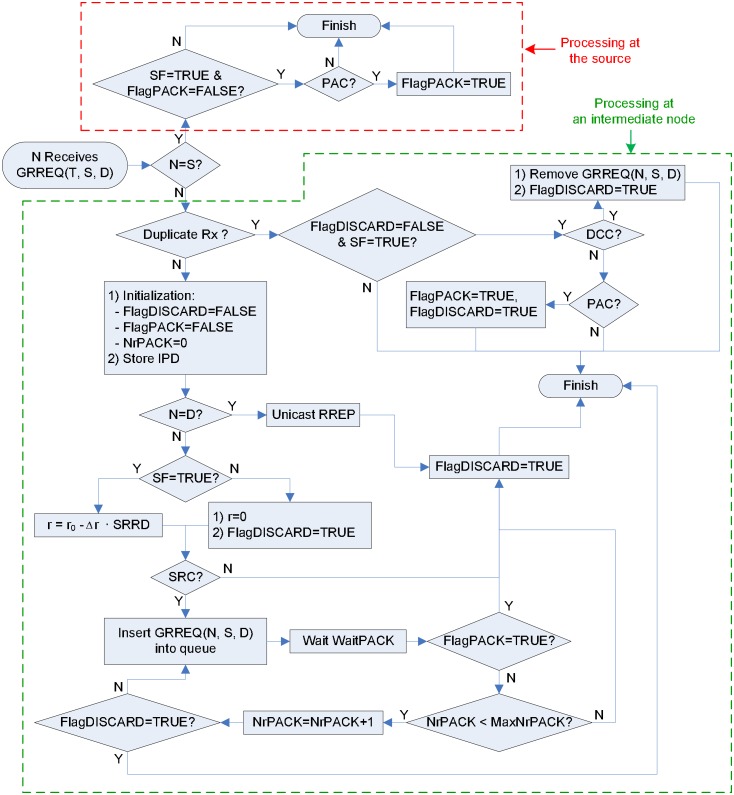
Flowchart of detailed procedure for the passive acknowledgement and the redundancy control of the GRREQ.

It is shown in Fig 8 how duplicate GRREQs can be parsed for the redundancy control and the passive acknowledgement of the GRREQ. In this figure, it is assumed for simple and clear presentation that destination only flag, which is D in RFC3561, is set to TRUE. However, it can be easily extended to include the case of de-activated D flag. One-bit flag FlagDISCARD, which is associated to a specific GRREQ, plays a crucial role to determine whether a duplicate GRREQ can be parsed at an intermediate node or not. A duplicate GRREQ can be parsed only when the FlagDISCARD is FALSE. The FlagDISCARD is set to TRUE if parsing duplicate GRREQ is not necessary any more or impossible. In Fig 8, SF and SRRD are obtained from the received GRREQ message. FlagPACK, NrPACK, MaxNrPACK, and WaitPACK, which are terms to manipulate the PAC, are the same as the GRREQ initiation procedure discussed in Appendix 3.2. The flow chart of Fig 8 is divided into two parts: (1) passive acknowledgement at the source, (2) redundancy control and passive acknowledgement at an intermediate node.

If the source receives a duplicate GRREQ, checks the PAC only when the SF is TRUE and the FlagPACK is FALSE. If the PAC is satisfied, the FlagPACK, which is appeared in Fig 7, is set to TRUE.

If an intermediate node N, which is not the source, receives GRREQ(*T, S, D*), check whether the received GRREQ is duplicate or not. If the GRREQ is heard for the first time, both the FlagDISCARD and the FlagPACK are set to FALSE, and the NrPACK is initialized to zero. If *N* is the destination, unicast the RREP and set the FlagDISCARD to TRUE. Otherwise, the GRREQ is rebroadcasted differently depending on the status of the SF. If the SF is TRUE, the GRREQ is selectively rebroadcasted. The value of *r* is calculated by Eq (14). If the SF is FALSE, the GRREQ is rebroadcasted non-selectively, i.e., the propagation of the GRREQ is similar to that of the RREQ in the AODV. In this case, the FlagDISCARD is set to TRUE because duplicate GRREQs can not be parsed any more. In addition, the value of *r* is set to zero, which removes the selective rebroadcast feature of the GRREQ and which avoids unnecessary counter-propagation of the GRREQ.

If *r* is determined, check whether the SRC is satisfied or not. If the SRC is not satisfied, the FlagDISCARD is set to TRUE because node *N* does not join the route discovery procedure. Otherwise, broadcast GRREQ(*N, S, D*). The repetition loop using the FlagPACK is similar to that of the GRREQ initiation procedure at the source, except for setting the FlagDISCARD to TRUE when the FlagPACK is still FALSE even after MaxNrPACK-times ReTx of the GRREQ. This activation of the FlagDISCARD is needed because the PAC check is not required any more.

If the received GRREQ is duplicate, this duplicate GRREQ can be parsed only when the SF is TRUE and the FlagDISCARD is FALSE. These two requirements for parsing duplicate GRREQ are named as RPD for convenience. If the RPD and the RCC are satisfied, we remove the corresponding GRREQ in the queue and set the FlagDISCARD to TRUE because N leaves the route discovery procedure. If the RPD is satisfied but not the RCC, we check the PAC. If the PAC is satisfied, both the FlagPACK and the FlagDISCARD are set to TRUE.

### 3.4 Example for the GRREQ propagation in a unicast manner

**Fig 9:**
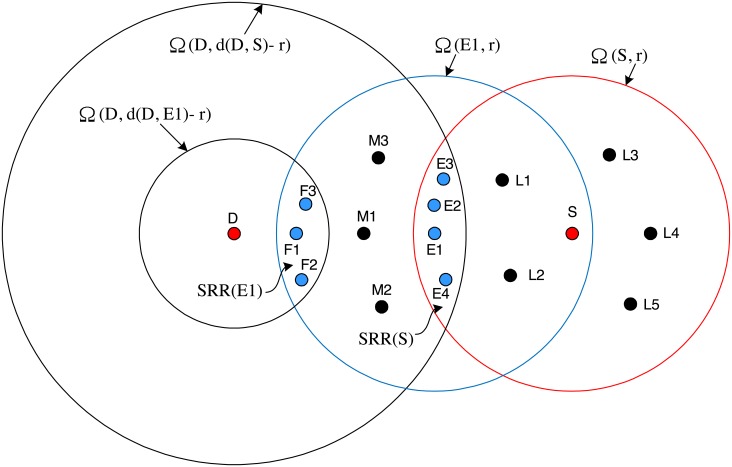
Example for the GRREQ propagation.

It is shown in Fig 9 how the GRREQ is propagated in a unicast manner. In this illustrative example, assuming perfect physical layer and data-link layer, we give step-by-step explanation of the redundancy control and the passive acknowledgement. The locations of nodes are summarized as follows,

- Group E: *Ei* ∈ Ω(*S*, *R*) ∩ Ω(*D*, d(*D, S*)-*r*), i.e., *Ei* ∈ SRR(*S*), ∀*i*.
- Group F: *Fi* ∈ Ω(*E1*, *R*) ∩ Ω(*D*, d(*D, E1*)-*r*), i.e., *Fi* ∈ SRR(*E1*), ∀*i*.
- Group L: *Li* ∈ Ω (*S*, *R*) and *Li* ∉ Ω (*D*, d(*D, S*)-*r*), i.e., *Li* ∉ SRR(*S*), ∀*i*.
- Group M: *Mi* ∈ Ω (*E1*, *R*) and *Mi* ∉ Ω (*D*, d(*D, E1*)-*r*), i.e., *Mi* ∉ SRR(*E1*), ∀*i*.

If *S* initiates route discovery procedure to find a route from *S* to *D*, chronological order of key events is given below:

1. S disseminates GRREQ(*S, S, D*).
2. Group L receives GRREQ(*S, S, D*) and group L discards GRREQ(*S, S, D*) because all nodes of group L are out of SRR(*S*). The FlagDISCARD of group L is set to TRUE. Group L is excluded from the route discovery procedure. If *r* is equal to zero (SF is FALSE), *L1* and *L2* broadcast GRREQ(*L1, S, D*) and GRREQ(*L2, S, D*), respectively, but *L3, L4*, and *L5* do not rebroadcast GRREQ(*S, S, D*) in spite of the deactivated SF, in which we can see the avoidance of the backward propagation of the GRREQ.
3. Group E receives GRREQ(*S, S, D*) and all nodes of group E are within SRR(*S*), which means that group E satisfies the SRC created by GRREQ(*S, S, D*). Therefore, each *Ei* inserts GRREQ(*Ei, S, D*) into queue and stores the IPD with respect to GRREQ(*S, S, D*).
4. *E1* broadcasts GRREQ(*E1, S, D*) for the first time among group E.
5. *E2, E3*, and *E4* receive GRREQ(*E1, S, D*), which is regarded as duplicate of GRREQ(*S, S, D*) at group E. The hop count of GRREQ(*E1, S, D*) is one, which is larger than that of GRREQ(*S, S, D*) by one. The hop count of GRREQ(*S, S, D*) is obtained from the stored IPD of *Ei*. Therefore, the RCC is satisfied by GRREQ(*E1, S, D*) at *E2, E3*, and *E4*. Now, *E2, E3*, and *E4* delete GRREQ(*E2, S, D*), GRREQ(*E3, S, D*), and GRREQ(*E3, S, D*) in queue, respectively. Eventually, *E2, E3*, and *E4* do not rebroadcast GRREQ(*S, S, D*) and are excluded from the route discovery procedure. The FlagDISCARD of *E2, E3*, and *E4* is set to TRUE due to the satisfied RCC.
6. *L1* and *L2* receive GRREQ(*E1, S, D*). Since the FlagDISCARD of *L1* and *L2* is TRUE from the second event, *L1* and *L2* immediately discard this duplicate GRREQ.
7. Group M receives GRREQ(*E1, S, D*). Group M discards GRREQ(*E1, S, D*) because group M is out of SRR(*E1*), which is similar to the second event. The FlagDISCARD of group M is set to TRUE.
8. *S* receives GRREQ(*E1, S, D*), which is regarded as duplicate of GRREQ(*S, S, D*). The hop count of GRREQ(*E1, S, D*) is one, which is larger than that of GRREQ(*S, S, D*) by one. Therefore the PAC at the source is satisfied by GRREQ(*E1, S, D*), and the FlagPACK, which is the passive acknowledgement at the source *S*, is set to TRUE.
9. Group F, which is located within SRR(*E1*), receives GRREQ(*E1, S, D*). Similar to the 3rd event, each *Fi* inserts GRREQ(*Fi, S, D*) into queue and stores the IPD with respect to GRREQ(*E1, S, D*).
10. *F1* broadcasts GRREQ(*F1, S, D*) for the first time among group F.
11. *F2* and *F3* receive GRREQ(*F1, S, D*). Similar to the 5th event, *F2* and *F3* delete GRREQ(*F2, S, D*) and GRREQ(*F3, S, D*) in queue, respectively. The FlagDISCARD of *F2* and *F3* is set to TRUE.
12. Group M receives GRREQ(*F1, S, D*). Since the FlagDISCARD of group M is TRUE from the 7th event, group M immediately discards this duplicate GRREQ.
13. *E1* receives GRREQ(*F1, S, D*), which is regarded as duplicate of GRREQ(*S, S, D*). *E1* can parse GRREQ(*F1, S, D*) because the FlagDISCARD of *E1* is FALSE. The hop count of GRREQ(*F1, S, D*) is two, which is larger than that of GRREQ(*S, S, D*) by two. The hop count of GRREQ(*S, S, D*) is obtained from the stored IPD of *E1*. Therefore, the RCC can not be satisfied by GRREQ(*F1, S, D*), but the PAC is satisfied by GRREQ(*F1, S, D*). The FlagPACK, which is the passive acknowledgement at the intermediate node E1, is set to TRUE. The FlagDISCARD is also set to TRUE due to the satisfied PAC.
14. *D* receives GRREQ(*F1, S, D*) and unicasts the RREP.

## 4 Simulation results

To demonstrate the performance of the proposed GAODV, we compare the GAODV with the original AODV through QualNet computer simulations. Through this comparison, the GAODV can be also indirectly compared with the other existing methods by examining their capabilities in reducing the overhead of the AODV.

### 4.1 Connectivity

The node density should be sufficiently high for the selective rebroadcast mechanism of the GRREQ to work. We present simulation results to show the required node density of the GAODV to guarantee a certain connectivity under the assumptions given in Section 2.4.

**Fig 10:**
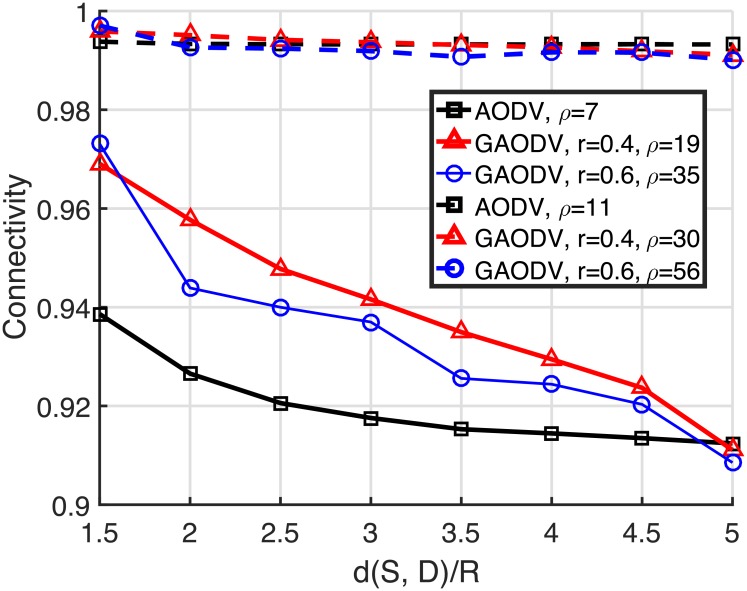
Required node density to achieve the connectivity of 0.9 and 0.99.

**Fig 11:**
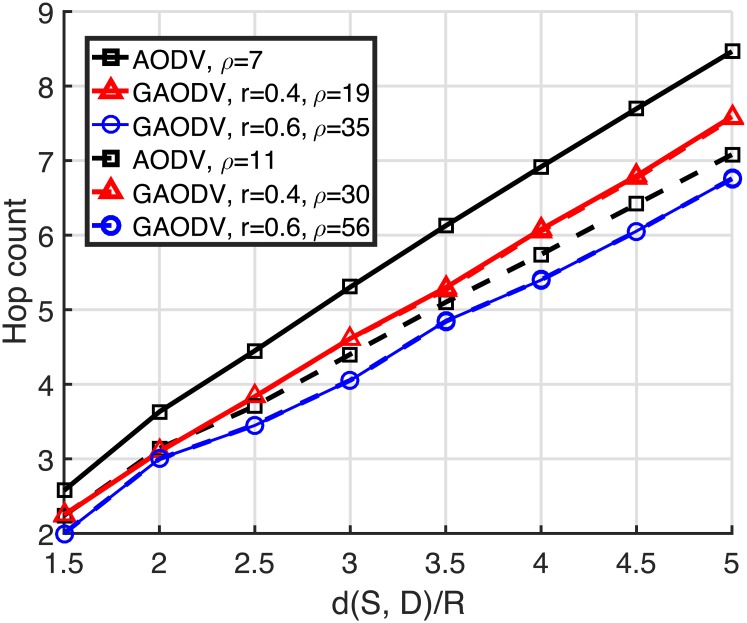
Hop count to achieve the connectivity of 0.9 and 0.99.

Figs 10 and 11 show the average of 10^5^ independent experiments for the required node density and the hop count to achieve the connectivity of 0.9 and 0.99. The hop count is counted only for the connected path, and the propagation of the GRREQ/RREQ is stopped as soon as the GRREQ/RREQ reaches the destination. The horizontal axis is the distance between the source and the destination normalized by *R*, and the range of this normalized distance is from 1.5 to 5. From Fig 10, to achieve the connectivity of 0.9, the node density of the AODV and the GAODV with *r* = 0.4 and *r* = 0.6 is required to be higher than 7, 19, and 35, respectively, which are analogous to the estimated theoritical values (5, 14, and 30, respectively) in Section 2.4. In this case, the required node density of the GAODV with *r* = 0.4 and *r* = 0.6 is greater than that of the AODV by 2.7- and 5-times, respectively. It is obvious that the required node density of the GAODV dereases as *r* decreases, but the decrease of *r* leads to the increase of the hop count as shown in Fig 11. However, the hop count increase is just less than one in Fig 11. It can be observed in Fig 11 that the hop count of the GAODV is not dependent on the node density, which shows that the average hop distance is determined by *r*. The observations discussed so far are also valid for the connectivity of 0.99. The hop count of the AODV with *ρ* = 7 is larger than that of the GAODV because the amount of zigzag is large due to the low node density. Note that the hop count of the AODV converges to a step function as the node density goes to infinity.

In order to verify Eqs (3) and (4), we compare the required node density of the GAODV with *r* = 0.4 and *r* = 0.6 at the distance of 1.3*R* as shown in Table 1. In this table, *ρ*_*req*,0.9_ and *ρ*_*req*,0.99_ are the required node density to achieve the connectivity of 0.9 and 0.99, respectively. The GRREQ propagation with *r* = 0.4 and *r* = 0.6 is completed in two hops at the distance of 1.3*R* and Eq (3) represents the analytic connectivity at this distance, which explains why we choose the distance of 1.3*R*. Any distance will do as long as it is chosen within the range of (*R*, 1.4*R*). The required node density obtained by the simulation is the smallest integer which exceeds the given connectivity. Table 1 shows a good match between the simulation and Eq (3) reinforcing the validity of the connectivity analysis. Also, it can be seen that the change of *ϵρ*_*req*,0.9_ and *ϵρ*_*req*,0.99_ obtained by the simulation is less than 3% in spite of nearly doubled *ϵ* due to the decrease of *r* from 0.6 to 0.4.

**Table 1:** Verification of Eqs (3) and (4) at the distance of 1.3*R*.

Item		GAODV, *r* = 0.4	GAODV, *r* = 0.6
*ϵ*		0.1825	0.0934
*ρ*_*req*,0.9_	Theory	11.4270	23.4828
	Simulation	12	24
*ρ*_*req*,0.99_	Theory	22.8539	46.9657
	Simulation	24	48
*ϵρ*_*req*,0.9_	Theory	2.0854	2.1933
	Simulation	2.1900	2.2416
*ϵρ*_*req*,0.99_	Theory	4.1708	4.3866
	Simulation	4.3800	4.4832

### 4.2 Imaginary destination

In this section, we demonstrate how the GAODV with the imaginary destination (GAODV-ID) helps reducing the uncertainty of the destination’s location. The GAODV-ID is compared with the original GAODV without the imaginary destination (GAODV-WID) using coverage ratio (CR) as a figure of merit. The CR is defined by the proportion of the area of Ω(*D*, *γ*) covered by GRREQ rebroadcasts. To avoid the interrupt of the GRREQ propagation due to imperfect connectivity, *ρ* is set to 100. In addition, *r* is fixed at 0.6.

**Fig 12:**
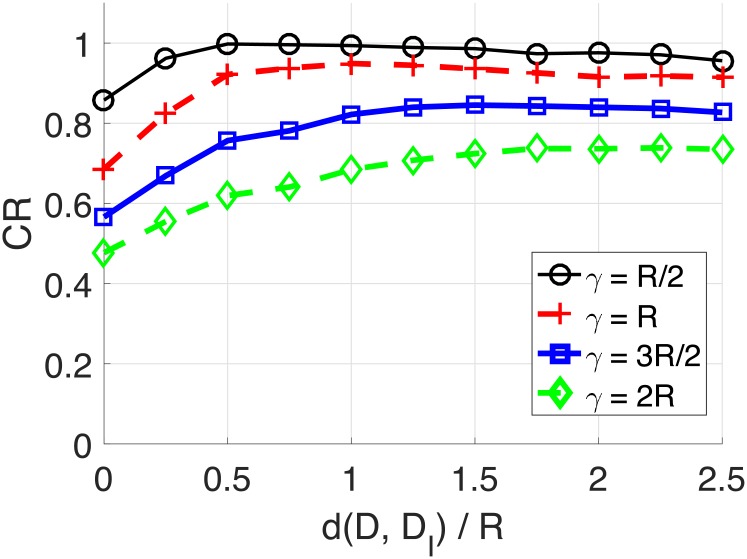
Coverage ratio for the change of the imaginary location: d(S, D) = 3*R*.

**Fig 13:**
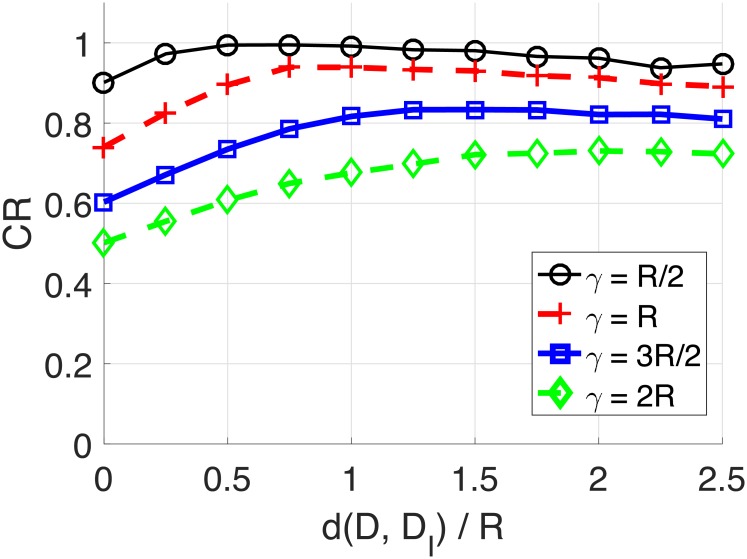
Coverage ratio for the change of the imaginary location: d(S, D) = 5*R*.

Figs 12 and 13 show the CR with respect to the imaginary destination and *γ*. The horizontal axis is d(*D, D_I_*)/*R*, where *D_I_* is the the imaginary location of the destination. The distance from the source to the destination is fixed at 3*R* in Fig 12. For each *γ*, the CR increases as d(*D, D_I_*) increases from 0 to *γ*, but the CR decreases if d(*D, D_I_*) is larger than *γ*. Therefore, the CR is maximum if d(*D, D_I_*) is the same as *γ*, which confirms the claim of Section 2.5. Furthermore, the optimum imaginary destination turns out to be independent of d(*S, D*) as shown in Fig 13. The only difference of Fig 13 and Fig 12 is that d(*S, D*) changes from 3*R* to 5*R*.

**Fig 14:**
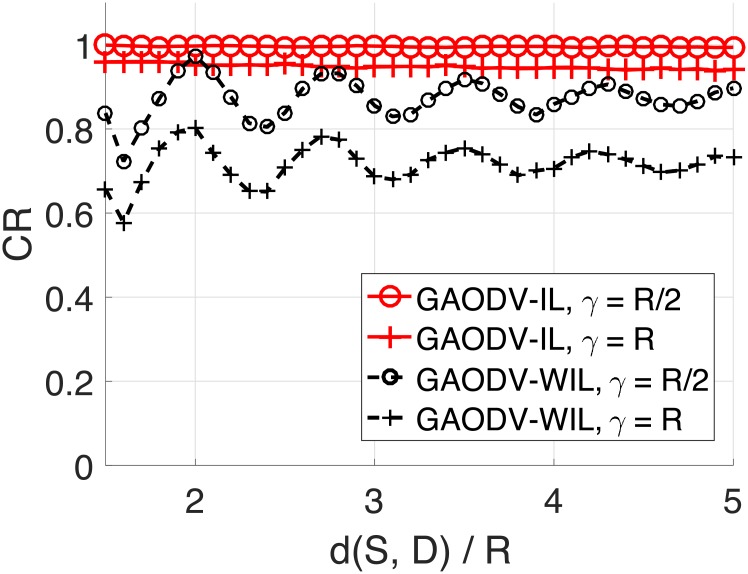
Coverage ratio for the change of the distance from the source to the destination: Mobility radius of *R*/2 and *R*.

**Fig 15:**
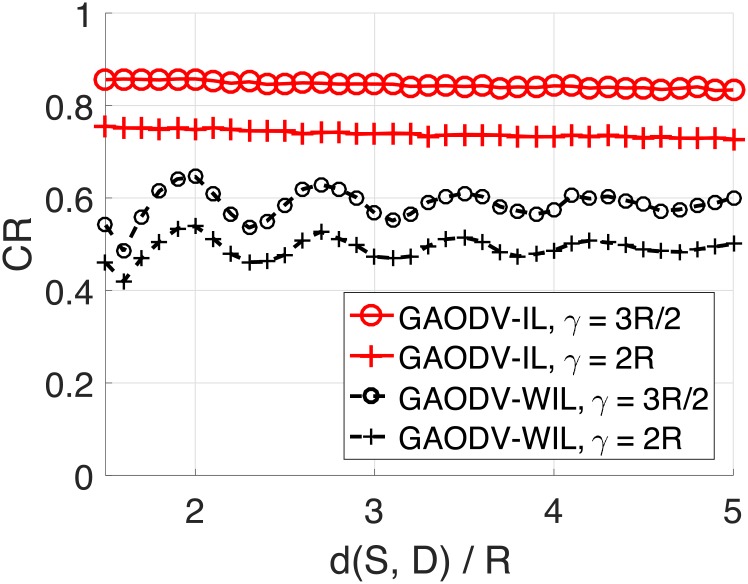
Coverage ratio for the change of the distance from the source to the destination: Mobility radius of 3*R*/2 and 2*R*.

Figs 14 and 15 show the CR for the change of d(*S, D*) and *γ*. The imaginary destination of the GAODV-ID is set to be the optimum location discussed in Section 2.5. The CR of the GAODV-ID decreases as d(*S, D*) increases, but the CR degradation of the GAODV-ID is relatively small. However, the CR of the GAODV-WID fluctuates with respect to d(*S, D*). The CR of the GAODV-WID is locally minimum at d(*S, D*) of 1.6*R* for all *γ*. In this case, all the nodes within SRR(*S*) are one-hop neighbors of *D* because *r* = 0.6, and the rebroadcast by a node within SRR(*S*) is the last hop of this GRREQ propagation in the GAODV-WID. If we recall Fig 6 which illustrates the aforementioned scenario, it can be seen why the CR of the GAODV-WID is significantly decreased at d(*S, D*) of 1.6*R*. The CR fluctuation of the GAODV-WID is reduced by the increase of d(*S, D*) due to the average effect by randomly chosen rebroadcast node.

**Fig 16:**
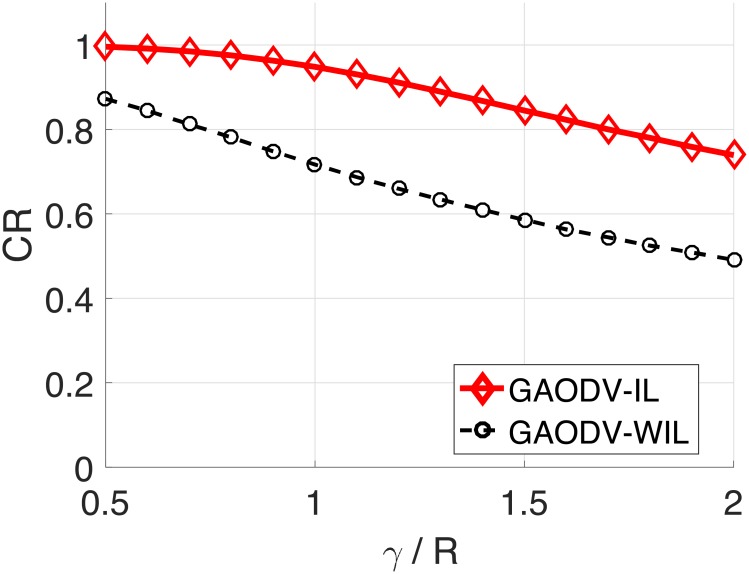
Average coverage ratio for the change of the mobility radius *γ*.

Fig 16 is the CR averaged over d(*S, D*) for the change of *γ*. The range of d(*S, D*) is from 1.5*R* to 5*R*. The CR of the GAODV-ID is 94.8% at *γ* of *R*, which is greater than that of the GAODV-WID by 23%. The CR gap between the GAODV-ID and the GAODV-WID is increased by the increase of *γ*. Considering that the maximum velocity of ship is less than 60 *km*/*h* and the communication range of marine VHF modem is typically dozens of kilometers, the CR loss of 5% of the GAODV-IL at *γ* = *R* may be acceptable in a maritime MANET.

### 4.3 Throughput, latency, and routing overhead

The routing overhead of the GAODV is not dependent on the start value of the time-to-live (TTL) because the GRREQ propagation stops if the destination’s location is included in the wireless communication area of an intermediate rebroadcast node. However, the expanded ring search (ERS) adopted in RFC3561 causes penalties in the AODV as follows:

- If the start value of the TTL is smaller than the required TTL, the RREQ should be retried until the TTL reaches this required TTL.
- If the start value of the TTL is larger than the required TTL, RREQ rebroadcasts which have the hop count larger than the required TTL are not necessary for the route discovery.
- In a high contention scenario, it can not be discriminated whether the route discovery fails due to the small TTL or not. In the worst case, the RREQ may be retried until a specified threshold TTL is reached.

For fair comparison, the TTL of the AODV is fixed at the value determined by the distance to the destination as follows,

$$\begin{array}{c} \text{TTL}=\lceil d(S,D)/R\rceil +1, \end{array}$$ (15)

where ⌈*x*⌉ is the smallest integer larger than or equal to *x*. The constant 1 in Eq (15) is to give an extra margin, which is sufficient in a dense network.

Simulation environments are given in Table 2. In our QualNet simulations, we try to organize a network topology that resembles maritime MANET. IEEE 802.11b radio model with the data rate of 2 Mbps is used as the physical layer. The MAC protocol is the distributed coordinate function (DCF) of IEEE 802.11. There are 1, 000 nodes within a two-dimensional area of 2,000 *m* × 1,250 *m*. The constant bit rate (CBR) data packet of 512 bytes is transmitted at the rate of 1 *packet*/*s*. Many nodes attempt to transmit this CBR packet to one sink node fixed at (0, 625). The number of CBR connections is denoted by *N*_*CBR*_. We use the random waypoint model to give mobility to the network. The minimum velocity (*v*_*min*_) is set to be zero, and the maximum velocity (*v*_*max*_) is changed from 5 *m*/*s* to 15 *m*/*s*. The performance measures are the packet delivery ratio (PDR), average end-to-end delay, and routing overhead. The routing overhead is represented by the number of RREQ/GRREQ rebroadcast normalized by the number of received data packets.

**Table 2:** Network environments in QualNet simulations.

Parameter		Value
Physical layer	Radio	IEEE 802.11b
	Data rate	2 Mbps
MAC		IEEE 802.11 DCF
Traffic	Type	CBR
	Size	512 bytes
	Interval	1 *s*
Mobility	Type	Random waypoint
	*v*_*max*_	5/10/15 *m*/*s*
	*v*_*min*_	0
	Pause time	0 *s*
Network dimension		2,000 *m* × 1,250 *m*
Number of nodes		1,000
*N*_*CBR*_		5, 10, 15, 20, 25
Routing		AODV, GAODV (*r* = 0.6)

**Fig 17:**
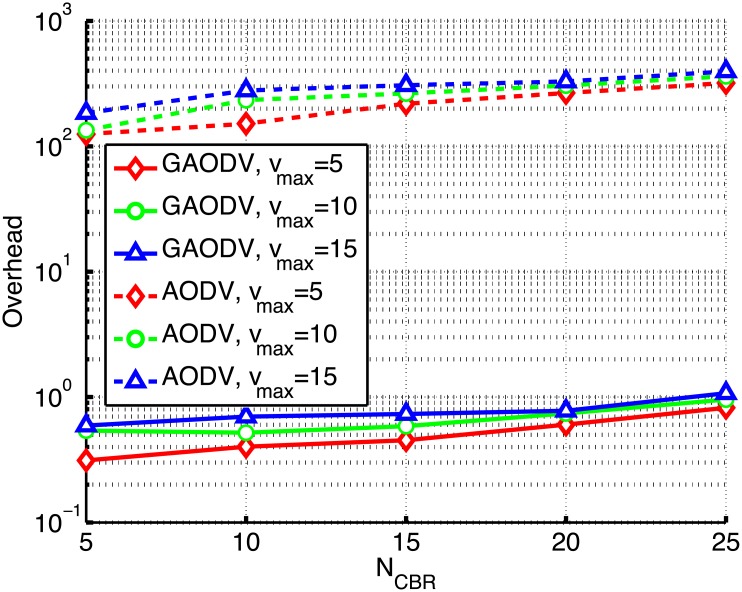
Routing overhead for the change of *N*_*CBR*_ and *v*_*max*_.

**Fig 18:**
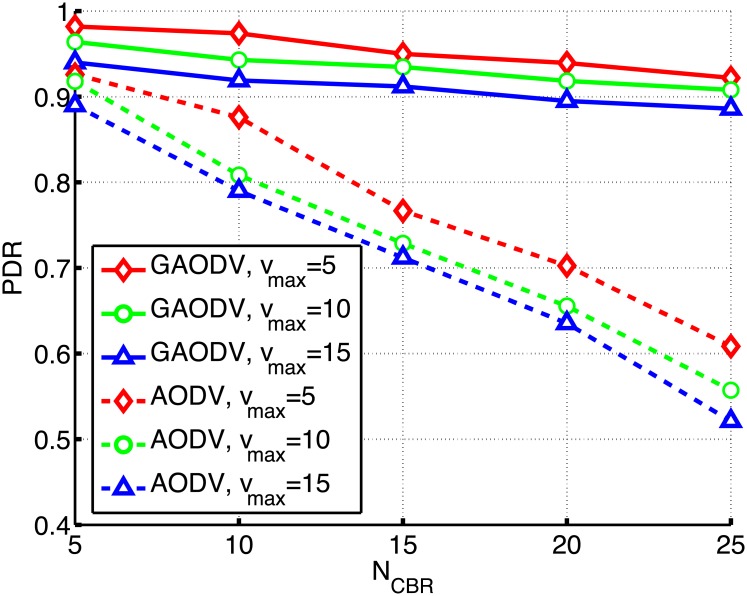
Packet delivery ratio for the change of *N*_*CBR*_ and *v*_*max*_.

**Fig 19:**
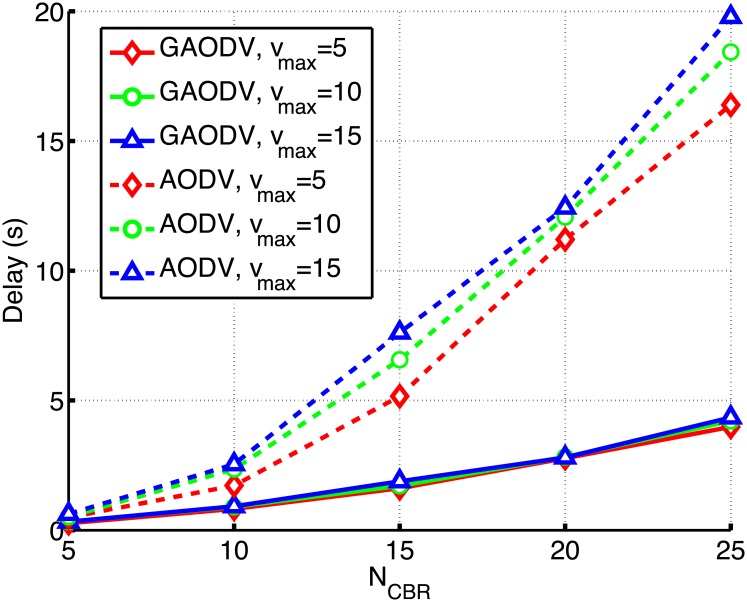
End-to-end delay for the change of *N*_*CBR*_ and *v*_*max*_.

We compare the proposed GAODV with the AODV according to the change of *N*_*CBR*_ and *v*_*max*_ as shown in Figs 17–19. In these figures, the solid and dotted lines are the plots of the GAODV and the AODV, respectively. In addition, the value of *v*_*max*_ can be clearly discriminated by the type of markers, that is the diamond, circle, and triangular markers correspond to *v*_*max*_ = 5*m*/*s*, *v*_*max*_ = 10*m*/*s*, and *v*_*max*_ = 15*m*/*s*, respectively. The key result is that the GAODV greatly outperforms the AODV in heavy traffic and high mobility scenarios. Fig 17 shows the routing overhead for the change of *N*_*CBR*_. It can be seen in Fig 17 that the routing overhead of the GAODV is less than that of the AODV by order of two or more. This greatly reduced routing overhead improves the PDR and the end-to-end delay in heavy traffic and high mobility scenarios as shown in Figs 18 and 19. Fig 19 illustrates the end-to-end delay averaged over all the received data packets.

**Fig 20:**
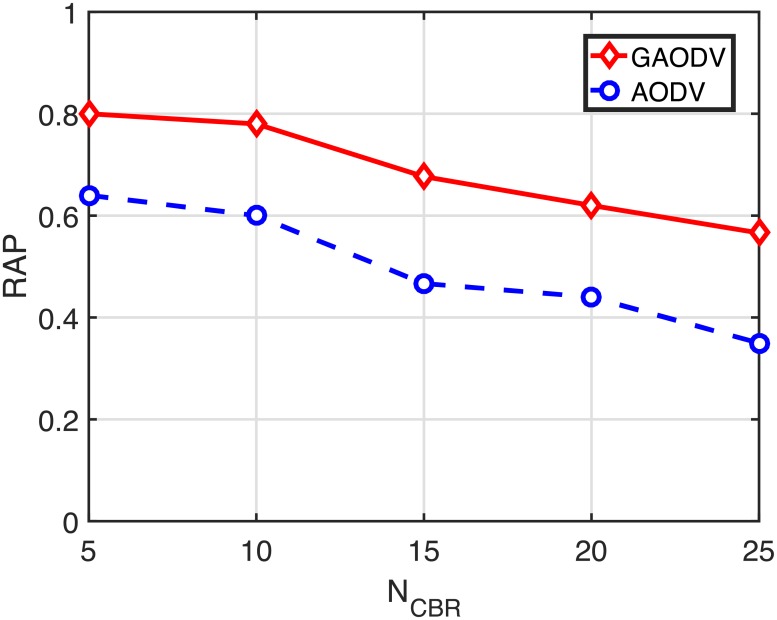
Route acquisition probability for the change of *N*_*CBR*_ when *v*_*max*_ = 5 *m*/*s*.

**Fig 21:**
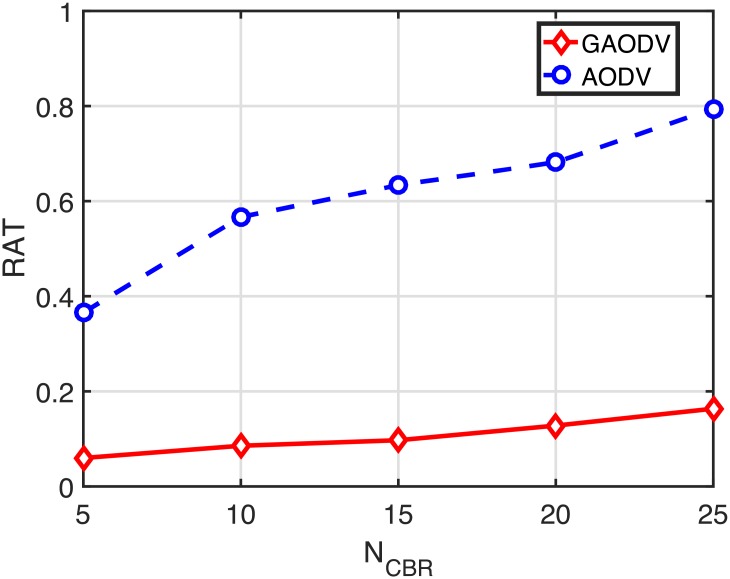
Route acquisition time for the change of *N*_*CBR*_ when *v*_*max*_ = 5 *m*/*s*.

At this point, we show how the route acquisition probability (RAP) and the route acquisition time (RAT) are closely related to the PDR and the end-to-end delay. In our simulation scenarios, *N*_*CBR*_ sources simultaneously start to find a route to the same destination. Therefore, initial route discovery period with large *N*_*CBR*_ results in a very high contention environment in the AODV. In the GAODV, however, this high contention period lies in a relatively low contention environment, which significantly improves the RAP and the RAT. Figs 20 and 21 show the RAP and the RAT in the case of *v*_*max*_ = 5*m*/*s*, respectively. To get the RAP and the RAT with only a single RREQ, the RREQ is not retried in Figs 20 and 21. The RAP of the GAODV is larger than that of the AODV by nearly 20%, and the RAT of the GAODV is just 1/4 of that of the AODV. Considering that each source starts to transmit data packets as soon as the RREP is received, the on-going sessions are adversely affected by the remained on-going route discovery procedures. In other words, data packets of many concurrent on-going sessions and RREQ packets compete with each other. These contentions increase the end-to-end delay of on-going sessions. Furthermore, active routes of on-going sessions can be damaged by these contentions, leading to route errors that initiate additional RREQs. These disadvantages experienced by data packets can be ignored in the GAODV because the number of GRREQ rebroadcasts is very small due to the unicast feature of the GRREQ propagation. However, in the AODV, although the RREQ packet length is relatively short compared to the data packet length, the number of RREQ rebroadcasts is significantly larger than the number of data packets of on-going sessions, which severely degrades the PDR and the delay performance. The advantages of the route discovery procedure of the GAODV can be summarized as follows:

- The unicast feature of the GRREQ propagation minimizes the collisions between route discovery procedures and on-going sessions.
- The higher RAP reduces overall attempts of route request.
- The fast route discovery shortens the transient high contention interval induced by concurrent route discovery procedures.

The PDR performance is shown in Fig 18. If *N*_*CBR*_ = 5 and *v*_*max*_ = 5 *m*/*s*, i.e., a low traffic scenario, the PDR of both the GAODV and the AODV is larger than 90% and the difference of the PDR between the GAODV and the AODV is less than 6%. However, if the traffic becomes heavier with the increase of *N*_*CBR*_, the performance gap between the GAODV and the AODV increases. If *N*_*CBR*_ increases to 25, the PDR of the GAODV is still larger than 90%, but the PDR of the AODV decreases to 60%. If *v*_*max*_ increases, the number of route errors also increases because of the increased topology change rate. If a route error is occurred in the GAODV, a new path is quickly provided without damaging the data packets of on-going sessions. Therefore, the data packet losses of the GAODV are approximately the same as the number of route errors, and the delay does not depend on *v*_*max*_ as shown in Fig 19, which verifies that the data packets are not influenced by the contention with the route discovery procedures induced by route errors. However, the data packets of on going sessions in the AODV should contend with the large number of RREQ packets more frequently in a heavier traffic and higher mobility scenario. Therefore, the number of data packet losses in the AODV is greater than the number of route errors, and the PDR drop is larger for larger *v*_*max*_ as shown in Fig 18. In addition, the delay of the AODV is greater for larger *v*_*max*_ as shown in Fig 19. The PDR degradation of the AODV due to the increased mobility is greater than that of the GAODV for all *N*_*CBR*_ in Fig 18. Specifically, if *v*_*max*_ increases from 5*m*/*s* to 15*m*/*s* when *N*_*CBR*_ is fixed at 25, the PDR of the GAODV drops by 3.6%, but the PDR loss of the AODV grows to 8.7%. In Fig 19, the delay of the AODV is less than twice that of the GAODV at the low traffic scenario with *N*_*CBR*_ of 5, and the delay of both the GAODV and the AODV is less than 1-*s*. Nevertheless, the delay of the AODV increases up to five times that of the GAODV as the traffic becomes heavier. We can conclude that the GAODV is less sensitive to the change of the mobility and the traffic than the AODV.

**Fig 22:**
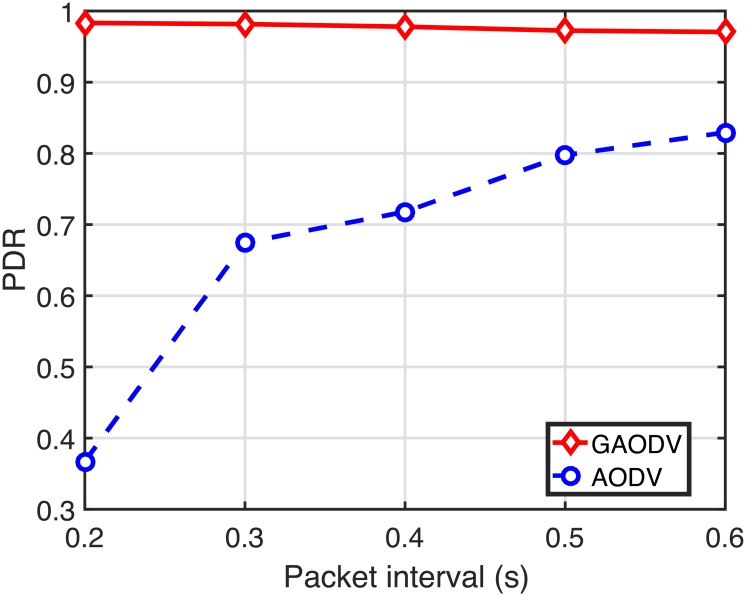
PDR for the change of the packet interval when *N*_*CBR*_ = 10 and *v*_*max*_ = 5 *m*/*s*.

**Fig 23:**
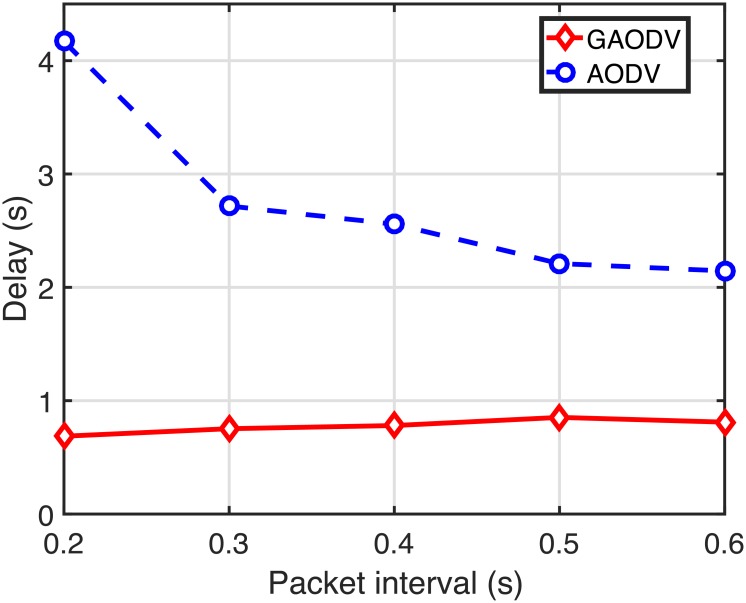
Delay for the change of the packet interval when *N*_*CBR*_ = 10 and *v*_*max*_ = 5 *m*/*s*.

**Fig 24:**
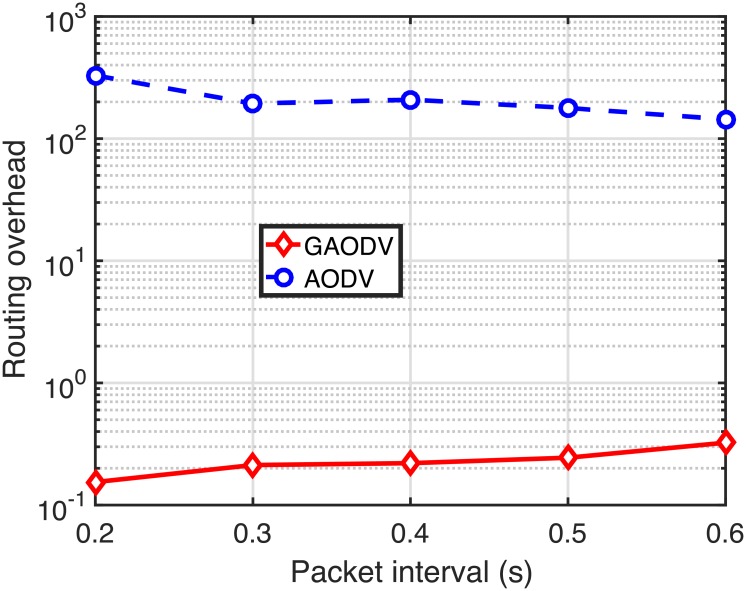
Routing overhead for the change of the packet interval when *N*_*CBR*_ = 10 and *v*_*max*_ = 5 *m*/*s*.

Figs 22–24 show the PDR, the delay, and the routing overhead, respectively, for the change of the packet interval. In these figures, *N*_*CBR*_ and *v*_*max*_ are fixed at 10 and 5 *m*/*s*, respectively. If the packet interval becomes shorter below the RAT in the AODV, more data packets of on-going sessions should compete with the large number of RREQ packets, which causes more route discovery failures and route errors. From Fig 21, the RAT of the GAODV is lower than 0.1-*s*, but the RAT of the AODV is about 0.6-*s*. Therefore, not unexpectedly, the PDR, the delay, and the routing overhead of the AODV are significantly degraded by these increased contentions between the data packets and the RREQ packets as shown in Figs 22–24. In Figs 22 and 23, the larger RAT of the AODV makes the PDR go below 40% at the packet interval of 0.2-*s*, and the delay becomes more than twice as the packet interval decreases from 0.6-*s* to 0.2-*s*. On the other hand, the PDR and the routing overhead of the GAODV are rather slightly improved by the decrease of the packet interval in Figs 22 and 24 because the number of route errors is nearly unchanged in spite of the decreased packet interval. Note that the topology change rate is not dependent on the packet interval, and the link broken due to the topology change is the main cause of the route errors in the GAODV. It can be concluded from Figs 22–24 that the GAODV can transmit at significantly higher packet rate than the AODV.

## Conclusion

We have proposed the GAODV that discovers a route in a unicast manner using the locations of the RREQ sender and the destination. The proposed GAODV has been implemented as a routing library of QualNet, and we conclude from QualNet simulations that the proposed GAODV can improve significantly the packet delivery ratio, the end-to-end latency, and the routing overhead of the AODV in a high density MANET. The required node density has been studied theoritically and verified using computer simulations. The GAODV is also applicable to fully mobile scenarios with the aid of the proposed imaginary destination method.
